# Short-term surgical outcomes of laparoscopy-assisted versus totally laparoscopic Billroth-II gastrectomy for gastric cancer: a matched-cohort study

**DOI:** 10.1186/s12893-017-0245-7

**Published:** 2017-04-21

**Authors:** Ji-Hyun Kim, Kyong-Hwa Jun, Hyung-Min Chin

**Affiliations:** Department of Surgery, St. Vincent’s Hospital, College of Medicine, The Catholic University of Korea, Seoul, Republic of Korea

**Keywords:** Gastric cancer, Billroth-II anastomosis, Laparoscopic gastrectomy, Surgical outcome

## Abstract

**Background:**

To evaluate feasibility and benefits of intracorporeal anastomosis, we compared short-term surgical outcomes between laparoscopy-assisted distal gastrectomy (LADG) and totally laparoscopic distal gastrectomy (TLDG) with Billroth-II (B-II) anastomosis for gastric cancer.

**Methods:**

Sixty patients underwent attempted B-II TLDG from 2011 through 2013. Patients who underwent B-II LADG prior to 2011 were matched to TLDG cases for demographics, comorbidities, tumor characteristics, and TNM stage. Perioperative and short-term surgical outcomes were compared between the two groups.

**Results:**

Clinicopathological characteristics of both groups were comparable. The B-II TLDG group had a shorter hospital stay (9.4 vs. 12.0 days, *P* = 0.038) and average incision size was smaller (3.5 vs. 5.4 cm, *P* = 0.030) than in the B-II LADG group. Anastomotic leakage was not recorded in either group, and there were no differences in the rates of perioperative complications and in inflammatory parameters between the two groups.

**Conclusions:**

This study suggests that B-II TLDG is feasible, compared to B-II LADG, and that it has several advantages over LADG, including a smaller incision, a shorter hospital stay, and more convenience during surgery. However, prospective randomized controlled studies are still needed to confirm that B-II TLDG can be used as a standard procedure for LDG.

## Background

The frequency of early detection of gastric cancer has increased due to recent advances in diagnostic techniques and widespread screening. Laparoscopy-assisted distal gastrectomy (LADG) for early gastric cancer is widely accepted because many clinical studies demonstrated its minimal invasiveness and comparable outcomes to those of open distal gastrectomy (ODG) [1, 2]. Moreover, several recent randomized controlled trials reported that LADG results in less estimated blood loss, fewer postoperative complications, and a shorter hospital stay than ODG [3, 4].

In LADG, lymph node dissection is performed laparoscopically. Subsequent resection and reconstruction of the stomach are performed extracorporeally, through a minilaparotomy. Most surgeons prefer LADG to totally laparoscopic distal gastrectomy (TLDG) because of the technical difficulties of intracorporeal anastomosis and concern over complications associated with anastomosis [5]. However, extracorporeal anastomosis in a narrow space with restricted vision is often problematic, particularly in obese patients with a thick abdominal wall and in those with a small remnant stomach. TLDG enables better visualization during anastomosis compared with LADG, overcoming those difficulties.

Goh et al. [6] first reported intracorporeal Billroth-II (B-II) gastrojejunostomy using laparoscopic linear staplers in 1992. Since then, advances in laparoscopic technique and instruments have made possible various totally laparoscopic anastomoses. Recent clinical studies of Billroth-I (B-I) gastroduodenostomy or Roux-en Y (R-Y) esophagojejunostomy have demonstrated that TLDG is safer, more technically feasible, and less invasive than LADG [7–9]. Meanwhile, the benefits of intracorporeal B-II anastomosis are often assumed and remain unproven. Only a few studies have reported the surgical outcomes of B-II TLDG [10, 11]. We therefore conducted this study to expand our experience and to perform a comparative analysis. B-II LADG patients were individually matched to B-II TLDG patients for demographics, tumor characteristics, and TNM stage, to assess the potential benefits of B-II TLDG.

## Methods

### Patients and matching process

The study was approved by the Institutional Review Board of St. Vincent’s Hospital (No. VC14RISI0107). Laparoscopic distal subtotal gastrectomy was restricted to patients whose tumors were clinical stage I. Sixty patients underwent B-II TLDG from 2011 through 2013. B-I or R-Y anastomoses were not included in this study. Matched-pair control patients were selected from 318 patients who underwent B-II LADG prior to the use of intracorporeal anastomosis (2008–2011). Candidates for controls were matched to TLDG cases for age, gender, comorbidities, body mass index (BMI), size and number of tumors, location of tumors, and TNM stage. First, tumor number and size were matched. After this first step of selection, the location of tumors and TNM stage were matched, and the LADG candidate who was best matched to each individual TLDG patient for demographics and comorbidities was selected. Peri- and postoperative outcomes were compared between the TLDG group (*n* = 60) and the matched-pair LADG group (*n* = 60).

### Surgical procedure

The operations were performed by two surgeons (HM and KH), who had each performed more than 300 laparoscopic procedures, including 60 laparoscopy-assisted gastrectomies, before this study. Under general anesthesia, patients were placed in the supine position with their legs apart and five trocars were used. Pneumoperitoneum was established using a Veress needle. One initial 11-mm trocar for the laparoscope was inserted below the umbilicus and four additional trocars were placed in the upper abdomen. In principle, laparoscopic lymph node dissection was performed in the same manner as the conventional open gastrectomy. All of the patients underwent at least D1+ or D2 lymph node dissection as described in the Japanese Classification of Gastric Carcinoma [12]. All of the dissecting procedures were performed using laparoscopic coagulation shears. Separation of the greater omentum from the transverse colon and dissection of the lymph nodes along the left gastroepiploic vessels (No. 4sb) was performed. Dissection was continued toward the pylorus, including the infrapyloric nodes (No. 6) and division of the right gastroepiploic vessels (No. 4d). The hepatoduodenal ligament was dissected and the suprapyloric nodes (No. 5) and the nodes along the common hepatic artery (No. 8) were dissected. Using a 60-mm Endo-GIA stapler (Covidien, Mansfield, MA), the duodenum was divided at a point 1 cm distal to the pylorus. Dissection of the nodes along the proper hepatic artery (No. 12a) and the proximal splenic artery (No. 11p) was performed when dissection of the D2 lymph node was necessary. After dissection of the nodes along the left gastric artery (No. 7) and celiac artery (No. 9), skeletonization of the lesser curvature of the gastroesophageal junction, with dissection of the right paracardial nodes (No.1) and the nodes along the lesser curvature (No. 3) was performed.

In LADG, epigastric incision was extended longitudinally to 4–5 cm in length and a wound protector was placed. The mobilized stomach was pulled out via the minilaparotomy. After resection of the distal part of the stomach using linear staplers, the proximal jejunum, 15–20 cm distal from Treitz’s ligament, was pulled out and gastrojejunostomy was performed using hand-sewn running sutures.

In TLDG, the stomach was divided using linear staplers, followed by removal of the resected specimen through the extended U-shaped skin incision below the umbilicus using a large plastic bag. The surgeon then checked the location of the tumor and the proximal cut margin on the removed specimen. If needed, additional gastric resection could be performed for oncological safety. However, there were no cases requiring additional resection among the patients enrolled in this study. Laparoscopic intracorporeal antecolic gastrojejunostomy and closure of the entry hole were performed using linear staplers.

### Postoperative data

Data related to patients’ clinicopathological characteristics, surgical procedures, and postoperative outcomes were collected retrospectively. In all of the cases, the disease was pathologically staged according to the 7th edition of the Union for International Cancer Control (UICC) TNM Cancer Staging Manual [13]. Clinicopathological characteristics of patients included age, sex, BMI, comorbidity, TNM stage, retrieved lymph nodes, tumor size, and tumor location. Surgical outcome parameters included the operative time, estimated blood loss, retrieved number of lymph nodes, intraoperative complications, incision size, time to first flatus, time to first soft diet, and duration of postoperative hospital stay. Postoperative complications were defined and graded according to the Claviend-Dindo surgical complication grading system [14]. Laboratory parameters, including white blood cell (WBC) count, neutrophil count, and CRP level at 1 and 4 days after surgery, were analyzed to compare the postoperative inflammatory response between the two groups. These data were compared between the two groups.

### Statistical analysis

Continuous data are presented as means ± SD and categorical data are presented as proportions. Statistical analyses were performed using Student’s *t*- test, the chi-squared test or Fisher’s exact probability test. A *P* value < 0.05 was considered statistically significant. All of the statistical analyses were performed using the Statistical Package for the Social Science (SPSS) version 20.0 for Windows (SPSS, Inc., Chicago IL).

## Results

### Patients’ demographics

Patient demographics (age, sex, BMI, and comorbidities), tumor characteristics (tumor size, number, and location), and TNM stage were comparable between TLDG and matched-pair LADG groups (Table 1). Because patients in the LADG group were selected from an earlier time period (before 2011) than the TLDG group (2011–2013), the median follow-up time was shorter in the TLDG group (median 23 months vs. 36.2 months, *P* = 0.045). By the criteria of the 7th edition of the UICC TNM Cancer Staging Manual, almost all of the patients (96.7%) were in stage I.

**Table 1:** Demographics of patients undergoing gastrectomy

	LADG (*n* = 60)	TLDG (*n* = 60)	*P* value
Age (yr)	60.9 ± 11.4	60.5 ± 12.1	0.889
Sex			
Male	40 (66.7)	40 (66.7)	1.000
Female	20 (33.3)	20 (33.3)	
BMI (kg/m^2^)	24.7 ± 2.8	24.1 ± 3.7	0.705
Comorbidity			
Diabetes	8 (13.3)	10 (16.7)	0.728
Cardiac	11 (18.3)	8 (13.3)	
Pulmonary	5 (8.3)	7 (11.7)	
Liver disease	3 (5.0)	2 (3.3)	
Cerebral infarction	0 (0)	1 (1.7)	
Tumor size	2.5 ± 1.4	2.8 ± 1.5	0.818
Tumor location			
Middle third	29 (48.3)	28 (46.7)	0.855
Lower third	31 (51.7)	32 (53.3)	
T stage			
T1	56 (93.3)	56 (93.3)	1.000
T2	3 (5.0)	3 (5.0)	
T3	1 (1.7)	1 (1.7)	
N stage			
N0	52 (86.7)	52 (86.7)	1.000
N1	8 (13.3)	8 (13.3)	
Stage (UICC 7th)			
I	58 (96.7)	58 (96.7)	1.000
II	2 (3.3)	2 (3.3)	

Data are presented as number (%) or mean ± standard deviation

### Surgical outcomes

Intraoperative surgical outcomes are shown in Table 2. Operative time was not significantly different between the groups (205.0 vs. 197.3 min, *P* = 0.381). For estimated blood loss, there was not statistically significant difference between two groups (100.5 vs. 117.2 mL, *P* = 0.056). There were no significant differences in time to flatus or start of soft diet. However, hospital stay and incision size in the TLDG group were significantly reduced compared to the LADG group. In obese patients (BMI ≥25 kg/m^2^), operation time was not statistically significant between two groups (218.2 ± 12.1 vs 201.7 ± 17.5 *P* = 0.054). There were no differences between the two groups in postoperative complications or in hospital stay (Table 3).

**Table 2:** Comparison of surgical outcomes between LADG and TLDG

	LADG (*n* = 60)	TLDG (*n* = 60)	*P* value
Operative time (min)	205.0 ± 22.4	197.3 ± 40.1	0.381
Open conversion	0	0	1.000
Intraoperative complications	0	0	1.000
Estimated blood loss (mL)	117.2 ± 81.6	100.5 ± 36.8	0.056
Retrieved lymph nodes	38.3 ± 11.4	39.4 ± 9.8	0.736
Laparotomy wound length	5.4 ± 1.3	3.5 ± 0.9	0.030
Time to first flatus (POD)	2.4 ± 1.2	2.1 ± 0.8	0.088
Time to starting soft diet (POD)	3.3 ± 1.8	3.1 ± 1.0	0.212
Postoperative hospital stay (days)	12.0 ± 3.5	9.4 ± 5.0	0.038

Data are presented as mean ± standard deviation

*POD* postoperative day

**Table 3:** Surgical outcomes in obese patients (BMI ≥25)

	LADG (*n* = 20)	TLDG (*n* = 20)	*P* value
Operation time	218.2 ± 12.1	201.7 ± 17.5	0.054
Postoperative complications	0	0	1.000
Postoperative hospital stay (day)	11.2 ± 3.6	10.4 ± 2.1	0.51

Data are presented as mean ± standard deviation

### Postoperative complications

Both TLDG and LADG had a complication rate of 8.3% (Table 4). Ileus with a paralytic obstruction pattern was observed on abdominal X-ray in two patients in the TLDG group and one patient in the LADG group. There was neither anastomotic leakage nor stricture in both groups. One patient in both groups suffered from duodenal stump leakage, which was managed conservatively. Anastomotic bleeding, which was resolved conservatively, occurred in one patient in the LADG group. Complications in other organ systems, such as the heart and lungs occurred in one patient in the TLDG group and two patients in the LADG group. The mortality rate in both groups was 0%.

**Table 4:** Comparison of postoperative complications between LADG and TLDG

	LADG (*n* = 60)	TLDG (*n* = 60)	*P* value
Overall postoperative morbidity	5 (8.3)	5 (8.3)	1.000
Type			
Paralytic ileus	1 (1.7)	2 (3.3)	0.308
Duodenal stump leakage	1 (1.7)	1 (1.7)	0.986
Anastomotic bleeding	1 (1.7)	0 (0)	0.308
Wound infection	0 (0)	1 (1.7)	0.320
Nonsurgical complications	2 (3.3)	1 (1.7)	0.571
Dindo-Clavien grade			
II	3 (5.0)	3 (5.0)	1.000
IIIa	2 (3.3)	2 (3.3)	1.000
Readmission	1 (1.6)	0 (0)	1.000
Reoperation	0 (0)	0 (0)	-
Hospital death	0 (0)	0 (0)	-

Data are presented as number (%)

### Inflammatory and nutritional parameters

The laboratory parameters are shown in Table 5. Inflammatory parameters, such as WBC count, neutrophil count, and CRP did not differ between groups at 1 and 4 days after surgery.

**Table 5:** Comparison of inflammatory markers between LADG and TLDG

	LADG (*n* = 60)	TLDG (*n* = 60)	*P* value
WBC count (10^9^/L)			
Preoperative	6.4 ± 1.7	6.5 ± 1.6	0.586
Postoperative day 1	10.3 ± 1.4	10.1 ± 1.2	0.250
Postoperative day 4	7.2 ± 1.2	6.9 ± 1.5	0.641
Neutrophil count (%)			
Preoperative	56.6 ± 7.4	55.3 ± 7.4	0.152
Postoperative day 1	82.7 ± 5.7	79.7 ± 3.3	0.224
Postoperative day 4	66.5 ± 5.6	62.6 ± 6.3	0.538
CRP (mg/L)			
Preoperative	0.6 ± 0.7	0.6 ± 0.8	0.250
Postoperative day 1	5.2 ± 2.9	4.8 ± 1.2	0.295
Postoperative day 4	7.7 ± 6.5	7.4 ± 5.6	0.792

Data are presented as or mean ± standard deviation

## Discussion

Conventionally, three gastrointestinal reconstructions after gastric resection—B-I, B-II, R-Y anastomosis—are performed in laparoscopic distal gastrectomy as well as in ODG [9, 15]. B-I anastomosis is the ideal reconstruction after gastrectomy in terms of maintaining physiological intestinal continuity and technical simplicity using a circular stapler. Therefore, many surgeons prefer this anastomosis compared to B-II or R-Y anastomosis during ODG and LADG. However, its application may be limited depending on tumor location and the size of the remnant stomach, because a remnant stomach of sufficient length is required to avoid tension in the anastomosis. In addition, delta-shaped anastomosis for intracorporeal gastroduodenostomy requires more precise laparoscopic manipulations than other types of reconstruction [16]. R-Y anastomosis can prevent reflux gastritis and esophagitis and reduces the likelihood of gastric cancer recurrence [17]. However, it is more complex and time-consuming than other types of anastomosis. Moreover, the extensive use of laparoscopic linear staplers can result in higher cost [18]. By comparison, one or two linear staplers are sufficient for intracoporeal gastrojejunostomy. B-II anastomosis is more easily applied in TLDG than B-I or R-Y anastomosis, irrespective of tumor location or of remnant stomach size [5, 19].

This study evaluated the feasibility, invasiveness, and benefit of B-II TLDG by comparing the short-term surgical outcomes in TLDG and LADG groups. In addition, this retrospective study involved a patient cohort matched 1:1 for age, sex, tumor characteristics, and TNM stage to minimize the effects of predisposing factors. Therefore, there were no differences in the baseline characteristics of patients in the two groups. We believe that this statistical method improved the accuracy of the comparison of short-term outcomes according to operative method.

As the number of reports that LADG is less invasive and provides faster recovery than ODG increases, the expectation that TLDG will also have these advantages over LADG has also increased. Indeed, several studies have compared TLDG and LADG. Song et al. [20] published a prospective, multicenter study, which showed that TLDG was more expensive but provided earlier bowel recovery than LADG and ODG. Ikeda et al. [21] reported that TLDG had several advantages over LADG including a smaller incision, less invasiveness, and better feasibility of a secure ablation. Kinoshita et al. [22] suggested that TLDG results in faster recovery, better cosmetic results, and improved quality of life in the short-term compared with LADG. Consistent with previous studies, our results showed that TLDG has advantages over LADG in terms of incision size and hospital stay. These findings suggest that B-II TLDG has better short-term outcomes than B-II LADG. In addition, there were no differences in the rates of postoperative and anastomosis-related complications between the TLDG and LADG groups. Large Korean and Japanese cohort studies have reported postoperative complication rates of 12.7% and 13.1%, respectively, for LADG [23, 24]. In this study, the postoperative complication rates were identical in the TLDG and LADG groups (5.8%). One case (1.7%) of anastomosis-related complications was found in the LADG group. Thus, we suggest that TLDG can be a safe and reliable procedure for gastric cancer.

We hypothesized that TLDG would be less invasive and be associated with improved postoperative inflammation and recovery of internal organs including the gastrointestinal tract, because, as well as a minilaparotomy at the epigastrium, pulling out of the stomach for extracorporeal anastomosis was not needed in TLDG, unlike LADG. Postoperative changes in WBC count, neutrophil count and CRP were determined to evaluate the inflammatory response. While several previous studies have reported lower WBC counts and CRP levels in TLDG compared with LADG [20, 21], our results showed no differences between groups. Therefore, additional studies using more sensitive inflammation markers, such as interlukin-6 (IL-6) and tumor necrosis factor (TNF) alpha are required to determine the superiority of TLDG in this respect.

In LADG, extracorporeal anastomosis is conducted in a limited working space with limited visual field, thus making it a difficult procedure, especially on obese patients. Extension of the laparotomy is necessary to obtain a better view for secure anastomosis on obese patients. In BMI > 25 kg/m^2^ patients, the operation time was shorter in the TLDG group than in the LADG group although it was not statistically significant. This finding indicates possibility that extracorporeal anastomosis needs more time because of the limited working space with restricted vision on obese patients. In this study, both TLDG and LADG were performed safely with few complications regardless of BMI. However, for obese patients, TLDG can provide more adequate working space with good visual field for the anastomosis.

Our study has several limitations. First, it was a retrospective study. Comparison between two groups was performed with limited data. More information could be collected if more variable biomarkers were used to examine, in particular, the relative invasiveness of the procedures. Second, the study size was small. However, this study was designed with a matched cohort. The enrolled patients were matched for age, sex, BMI, comorbidities, and tumor characteristics, which we would expect to compensate somewhat for its small size. Third, the surgeon’s learning curve may influence the data of this study, as enrollee selection depended upon the time period when the surgery was performed. However, as mentioned above, the surgeons already had significant experience in laparoscopic gastrectomy prior to the cases enrolled in this study. Also, as TLDG involves the same procedures as LADG for radical gastrectomy, with lymph node dissection preceding anastomosis, we believe that the effect of different operative periods should not be significant.

## Conclusion

In this study, surgical outcomes of B-II TLDG showed it to be feasible compared with those of B-II LADG. TLDG has several advantages over LADG, such as a smaller incision, a shorter hospital stay, and more convenience during surgery. However, prospective randomized controlled studies are still needed to confirm that B-II TLDG can be used as a standard procedure for LDG.

## Abbreviations

B-I: Billroth-I
B-II: Billroth-II
BMI: Body mass index
LADG: Laparoscopic-assisted distal gastrectomy
ODG: Open distal gastrectomy
R-Y: Roux-en-Y
SD: Standard deviation
TLDG: Totally laparoscopic distal gastrectomy
